# Significant potential of melatonin therapy in Parkinson’s disease – a meta-analysis of randomized controlled trials

**DOI:** 10.3389/fneur.2023.1265789

**Published:** 2023-10-10

**Authors:** Sadaf Iftikhar, Hafiz Muhammad Sameer, Zainab

**Affiliations:** Department of Neurology, King Edward Medical University/Mayo Hospital, Lahore, Pakistan

**Keywords:** melatonin, Parkinson’s disease, movement disorders, motor symptoms, sleep disturbances

## Abstract

**Objective:**

Since its discovery as an antioxidant, melatonin has been increasingly recognized for its therapeutic potential beyond sleep disturbances in neurodegenerative disorders. This study aims to evaluate efficacy of various melatonin doses, treatment durations, and formulations, in alleviating motor symptoms and sleep disturbances in Parkinson’s disease, the second most common neurodegenerative disorder worldwide.

**Methods:**

PubMed, Cochrane Library, ClinicalTrials.gov and other databases were systematically searched to retrieve randomized controlled trials (RCTs) administrating melatonin to Parkinson’s disease patients until June 10th, 2023. Outcomes including Unified Parkinson Disease Rating Scale (UPDRS) scores and Pittsburgh Sleep Quality Index (PSQI) scores, were pooled and reported as mean differences (MD) with 95% confidence intervals (CIs). Meta-analysis was performed using an inverse variance random-effects model in Review Manager 5.4 software. Trial Sequential Analysis was performed to avoid false-positive results from random errors.

**Results:**

Five RCTs with a total of 155 patients were included. Statistically significant reductions in UPDRS total scores were observed in groups receiving Melatonin ≥10 mg/day (MD = −11.35, 95% CI: −22.35 to −0.35, I^2^ = 0%, *p* = 0.04) and immediate release formulations (MD = −11.35, 95% CI: −22.35 to −0.35, I^2^ = 0%, *p* = 0.04). No significant effects on individual UPDRS II, III, and IV scores were observed, regardless of melatonin dosage and treatment duration. Moreover, significant improvements in PSQI scores were observed with only immediate-release melatonin formulations (MD = −2.86, 95% CI: −4.74 to −0.97, I^2^ = 0%, *p* = 0.003).

**Conclusion:**

Melatonin ≥10 mg/day for a minimum duration of ≥12 weeks in immediate-release formulations consistently demonstrated significant therapeutic potential in improving motor symptom and sleep disturbances in Parkinson disease. However, further trials are warranted to investigate its impact when initiated early in the disease course to fully explore its true therapeutic potential.

**Systematic review registration:**

Unique identifier: CRD42023427491 (PROSPERO).

## 1. Introduction

N-Acetyl-5-methoxytryptamine, commonly known as melatonin, is a neurohormone synthesized and secreted by the pineal gland (1, 2). While renowned for its pivotal role in regulating circadian cycles (3) and sleep–wake harmony (4), its identification as a powerful direct free radical scavenger (5–7) and an indirect antioxidant (8–15) have emerged as an unexpected revelation. Since its discovery as an antioxidant, melatonin has been increasingly recognized for its therapeutic potential beyond sleep disturbances across a wide range of medical conditions, specifically age-related neurodegenerative conditions (16–20). These neurodegenerative disorders, often driven by free radical-mediated apoptosis (21), present a challenge to treat due to limited blood–brain barrier penetration by various antioxidants. Exogenous melatonin, known for its ability to readily cross the blood–brain barrier (22, 23) coupled with its free radical scavenging and antioxidant properties, positions itself as a promising therapeutic agent for these conditions (24). While melatonin holds value for managing various neurodegenerative conditions, this article focuses only on its role in Parkinson’s disease, the second most common neurodegenerative disease worldwide (25).

Parkinson’s disease is a movement disorder characterized by progressive loss of dopaminergic neurons in substantia nigra pars compacta (25). Recent developments emphasize that, in addition to α-synuclein-induced cytotoxicity (26), increased free radical production, oxidative stress and neuroinflammation also contribute to this neuronal degeneration (27–30). Melatonin’s ability to inhibit α-synuclein-induced cytotoxicity (31), along with its free radical-neutralizing and antioxidant properties (32–34) offer potential for attenuating this neuronal loss. Furthermore, sleep disturbances, the most disabling non-motor symptoms in Parkinson disease (35, 36), are associated with reduced expression of melatonin receptors in the substantia nigra (37). Additionally, a drop in endogenous melatonin concentrations is also observed following Antiparkinson therapy (38). Exogenous melatonin can effectively counteract these reductions, and its sleep-inducing effects (4, 39, 40), along with its safe profile, make it superior for addressing sleep disturbances compared to alternatives like Clonazepam, with undesirable hangover effects (41). Although, ample experimental evidence (18, 19, 31, 33, 42) supports melatonin use in Parkinson’s disease, further evaluation is necessary to determine its real-world clinical applications.

Randomized controlled trials (RCTs) exploring melatonin’s efficacy in Parkinson’s disease have yielded inconsistent results, warranting a meta-analysis. However, only existing meta-analysis (43) evaluates melatonin exclusively for sleep disorders in Parkinson’s disease. It also fails to stratify the analysis by doses, treatment durations, and melatonin formulations, such as immediate-release (half-life: 20–30 min) and prolonged-release (half-life: 3–4 h) (44). This limited approach emphasizes the need for a newer updated and a more thorough meta-analysis. Therefore, this analysis aims to evaluate efficacy of various melatonin doses, treatment durations, and formulations compared to a placebo, in alleviating both motor symptoms and sleep disturbances in Parkinson’s disease, utilizing the standard Unified Parkinson Disease Rating Scale (UPDRS) (45, 46) and the Pittsburgh Sleep Quality Index (PSQI) (47).

## 2. Methods

This meta-analysis adheres to Preferred Reporting Items for Systematic Reviews and Meta-Analyzes (PRISMA) guidelines (48) and is registered with International Prospective Register of Systematic Reviews, PROSPERO (CRD42023427491).

### 2.1. Literature search

A comprehensive literature search was conducted from 1 June 2023 to 10 June 2023. Following databases were searched: MEDLINE (via PubMed), Cochrane Controlled Register of Trials (CENTRAL) (via Cochrane Library), ResearchGate, Google Scholar, Europe PMC, ClinicalTrials.gov, WHO ICTRP, and SciELO, without restrictions on language or publication period. Search terms included “Melatonin,” “melatonin therapy,” “melatonin supplement,” “Parkinson’s disorder,” and ‘Parkinsonism.’ among others. The detailed search strategy can be found in Supplementary Table S1. Additionally, the reference lists of previous meta-analyzes and included trials were manually screened to minimize the possibility of overlooking relevant studies.

### 2.2. Eligibility criteria and study selection

Studies were deemed eligible if they confirmed the following criteria: (1) Population: Participants were adults (age > 18), diagnosed with Parkinson’s disease using a recognized criterion (49); (2) Intervention: Experimental group received melatonin; (3) Comparator: Control group received a placebo; (4) Outcomes: Motor symptoms were assessed using the Unified Parkinson Disease Rating Scale (UPDRS) (45), or sleep quality was assessed using the Pittsburgh Sleep Quality Index (PSQI) (47); and (5) Study design: Either parallel-group RCTs or cross-over RCTs that reported pre-cross-over results to ensure the absence of residual effects from previous interventions. Exclusion criteria comprised: (1) Animal studies, case reports, nonrandomized trials or observational studies; (2) Trials with control group receiving drugs other than a placebo; and (3) Trials lacking eligible outcome measurements.

### 2.3. Data extraction

Electronic search results were imported into Mendeley Desktop. Two investigators (H and Z), blinded to each other’s decision, screened records independently. Redundant and unrelated records were removed by reviewing titles and abstracts. Full texts of remaining records were assessed for eligibility. Investigators used a pre-designed data collection sheet to extract: (1) Baseline characteristics; (2) UPDRS subscale and total scores; and (3) PSQI scores. Continuous variables were recorded as means with standard deviations (SDs), and when medians and ranges were reported, approximations were made based on Wan and Luo’s methods (50, 51). Corresponding authors were contacted, if necessary, to obtain missing data or additional details.

### 2.4. Risk of bias assessment in individual studies

The revised Cochrane “Risk of Bias” tool for RCTs (RoB 2.0) (52) was used to assess bias in individual studies. Articles were evaluated across five domains: randomization process, deviations from intended interventions, missing outcome data, outcome measurement and selection of reported result. Each item was categorized as low, some concerns, or high risk. Disagreements were resolved through discussion and reasoning, with a final decision made by a third independent reviewer (DS) if needed.

### 2.5. Confidence in cumulative evidence

Quality of evidence was assessed according to GRADE (grading of recommendations assessment, development and evaluation) approach using GRADEpro GDT software (53). Evidence was graded as high, moderate, low, or very low based on the proximity of true effect to the estimated effect. High grade indicates close proximity, moderate indicates likelihood of proximity with some possibility of difference, low indicates likelihood of potential difference, and very low indicates substantial difference.

### 2.6. Statistical analyzes

Measurements (UPDRS and PQSI scores) were treated as continuous outcomes. Mean changes were expressed as mean difference (MD) at a 95% confidence interval. Meta-analysis was performed using an inverse variance random-effects model. Subgroup analyzes was based on treatment duration, melatonin dose, and formulation. Statistical analysis was performed using Review Manager (RevMan, Version 5.4). Heterogeneity and variability were assessed using Chi^2^ and I^2^ statistics, with an I^2^ value above 50% indicating significant heterogeneity. To explore the causes of heterogeneity, a leave-one-out sensitivity analysis was performed. Due to small number of eligible studies (<10), funnel plots to assess publication bias could not be constructed (54). Still, Trial Sequential Analysis was employed to eliminate false-positive results caused by random errors (55).

## 3. Results

### 3.1. Study selection

**Figure 1:**
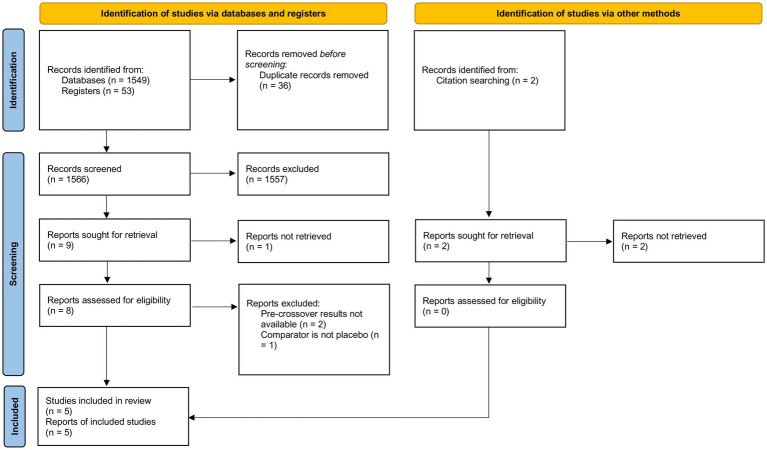
PRISMA 2020 flow diagram.

An in-depth database search yielded 1,602 records, while 2 articles were identified through additional sources. After removing duplicates (*n* = 36), primary screening excluded 1,557. Full-text screening of the remaining records, in accordance with inclusion criteria, resulted in the selection 5 RCTs for quantitative synthesis. The PRISMA flowchart is summarized in Figure 1.

### 3.2. Study and patient characteristics

Extensive literature review identified 5 RCTs spanning from 2007 to 2020 (56–60). Pooled sample included 155 patients, 75 (48.38%) in melatonin group and 80 (51.61%) in placebo group. Parkinson’s disease diagnosis was based on the UK Parkinson’s Disease Society Brain Bank criteria (49), except in one study (59) that used presence of at least 2 of the following: tremor, rigidity, and/or akinesia. In each trial different melatonin doses were used. Two trials (58,60) used prolonged-release melatonin (PRM), while, the rest employed immediate-release formulations. Trial durations also varied, two (59, 60) lasted 4 weeks, two (57, 58) lasted 12 weeks, and one (56) lasted 1 year.

UPDRS and PQSI scores were reported inconsistently. Across all RCTs, only 1 cohort (57) reported UPDRS I score, 2 cohorts (57, 59) reported UPDRS II scores, 4 cohorts (57–60) reported UPDRS III scores, 2 cohorts (57, 59) reported UPDRS IV scores, 3 cohorts (56–58) reported total UPDRS scores, and 4 cohorts (57–60) reported PQSI scores. Table 1 summarizes these characteristics of included RCTs.

**Table 1:** Study demographics of included RCTs.

Study (year)	Study design	Country	Participants		Melatonin (Intervention)			Comparator	Disease duration (years)^a^		Outcome measured	
			*N*	Diagnostic criteria	Dose/day	Formulation	Duration		Melatonin group	Placebo group	Disease severity	Sleep quality
Ortiz et al. (56)	Prospective double-blinded randomized clinical pilot trail	Mexico	13	UK Parkinson’s Disease Society Brain Bank criteria	50 mg	Immediate release	52 weeks	Placebo	Recently diagnosed	Recently diagnosed	UPDRS Total scores	
Daneshvar Kakhaki et al. (57)	Randomized, double-blind, placebo-controlled clinical trial	Iran	60	UK Parkinson’s Disease Society Brain Bank criteria	10 mg	Immediate release	12 weeks	Placebo	5.7 ± 1.9	5.5 ± 2.1	UPDRS I scores UPDRS II scores UPDRS III scores UPDRS IV scores UPDRS Total scores	PSQI score
Gilat et al. (58)	Phase-II, randomized, double-blind, placebo-controlled, parallel-group trial	Australia	30	UK Parkinson’s Disease Society Brain Bank criteria	4 mg	Prolonged release (PRM)	12 weeks	Placebo	5.07 ± 3.9	6.13 ± 4.4	UPDRS III scores UPDRS Total scores	PSQI score
Medeiros et al. (59)	Randomized, double-blind, parallel-group, placebo-controlled study	Brazil	18	Presence of at least 2 of following: tremor, rigidity and/or akinesia	3 mg	Immediate release	4 weeks	Placebo	6.40 ± 2.5	7.70 ± 6.5	UPDRS II scores UPDRS III score UPDRS IV scores	PSQI score
Ahn et al. (60)	Randomized, double-blind, placebo-controlled, multi-center trial	South Korea	34	UK Parkinson’s Disease Society Brain Bank criteria	2 mg	Prolonged release (PRM)	4 weeks	Placebo	5.0 ± 5.9	4.2 ± 4.4	UPDRS III scores	PSQI score

a Data presented as mean ± SD.

### 3.3. Quality assessment of included studies

**Figure 2:**
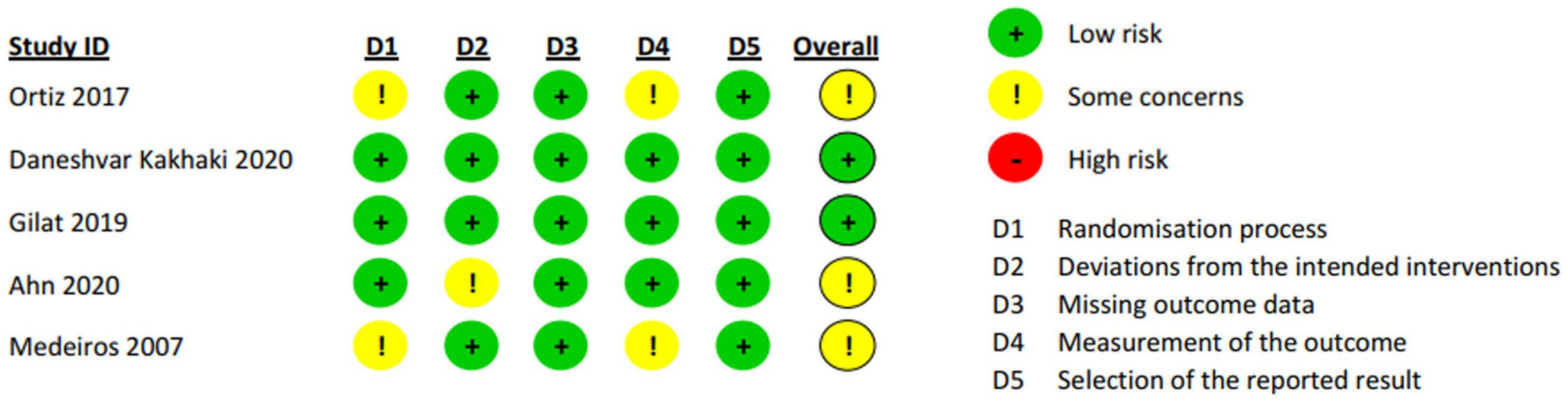
Quality assessment of included RCTs using RoB 2.0.

No trial was judged to have high risk of bias. Two trials (56, 59) raised concerns about overall bias due to insufficient explanation of randomization and allocation concealment methods, as well as inadequate blinding of outcome assessors. One trial (60) had concerns regarding insufficient blinding of carers and intervention providers (Figure 2). The remaining trials had low risk of bias in all domains.

### 3.4. Outcome

#### 3.4.1. Parkinson disease severity

**Figure 3:**
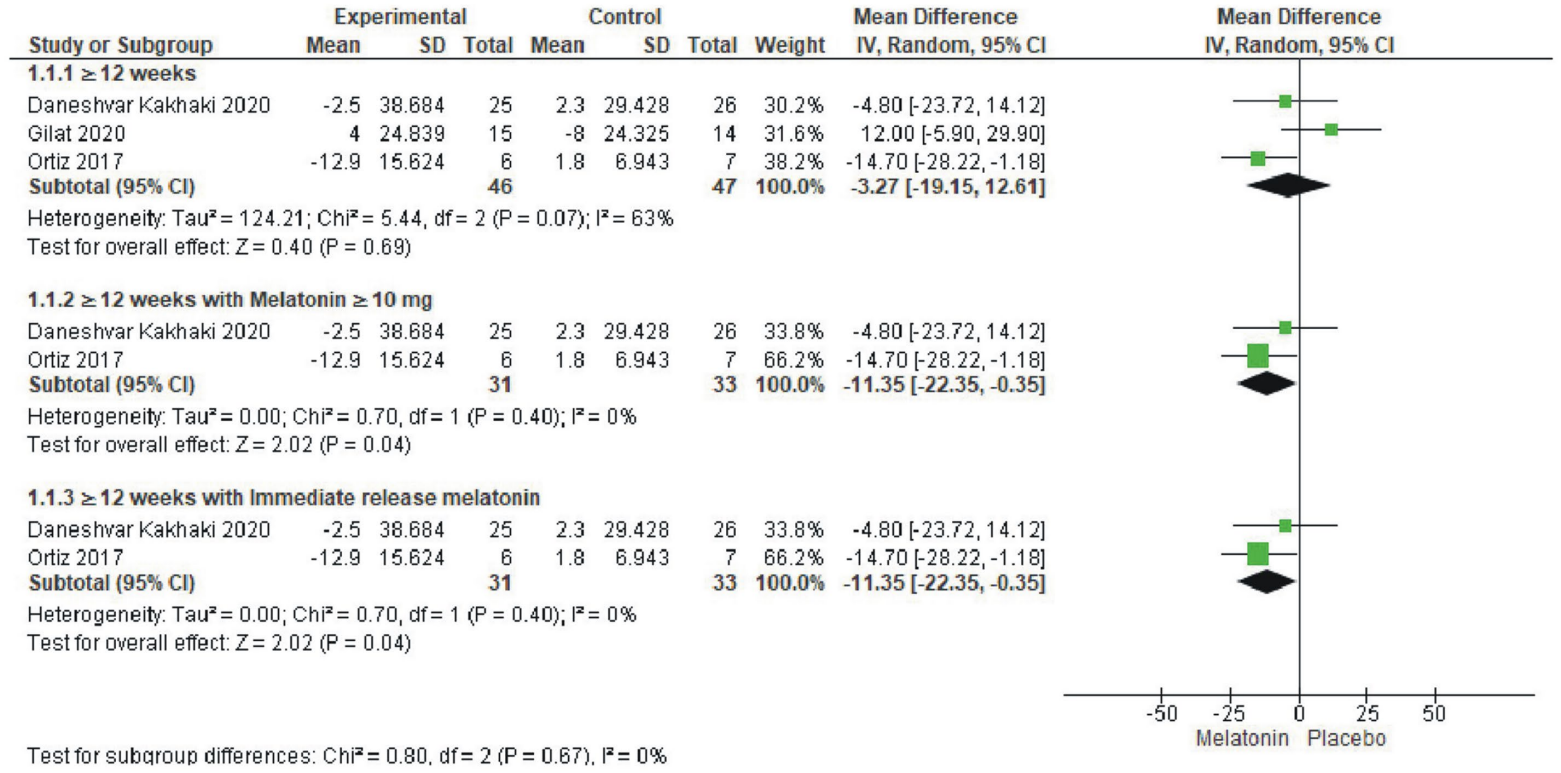
Forest plot comparing effects of exogenous melatonin on UPDRS total score.

Among the 5 included trials, data on UPDRS total scores were available in 3 trials involving 93 patients (56–58). Significant reductions in UPDRS total scores were observed in groups receiving Melatonin ≥10 mg/day (MD = -11.35, 95% CI: −22.35 to −0.35, I^2^ = 0%, *p* = 0.04) and immediate release formulation (MD = -11.35, 95% CI: −22.35 to −0.35, I^2^ = 0%, p = 0.04). However, no significant reduction and high heterogeneity was seen with Melatonin ≥4 mg/day (MD = −3.27, 95% CI: −19.15 to 12.61, I^2^ = 63%, *p* = 0.69, Figure 3). Sensitivity analysis in the Melatonin ≥4 mg/day group (I^2^ = 87%) eliminated heterogeneity upon removing the Gilat et al. trial from the analysis with the pooled estimate favoring melatonin significantly (MD = -11.35, 95% CI: −22.35 to −0.35, I^2^ = 0%, *p* = 0.04) (Supplementary Table S2).

**Figure 4:**
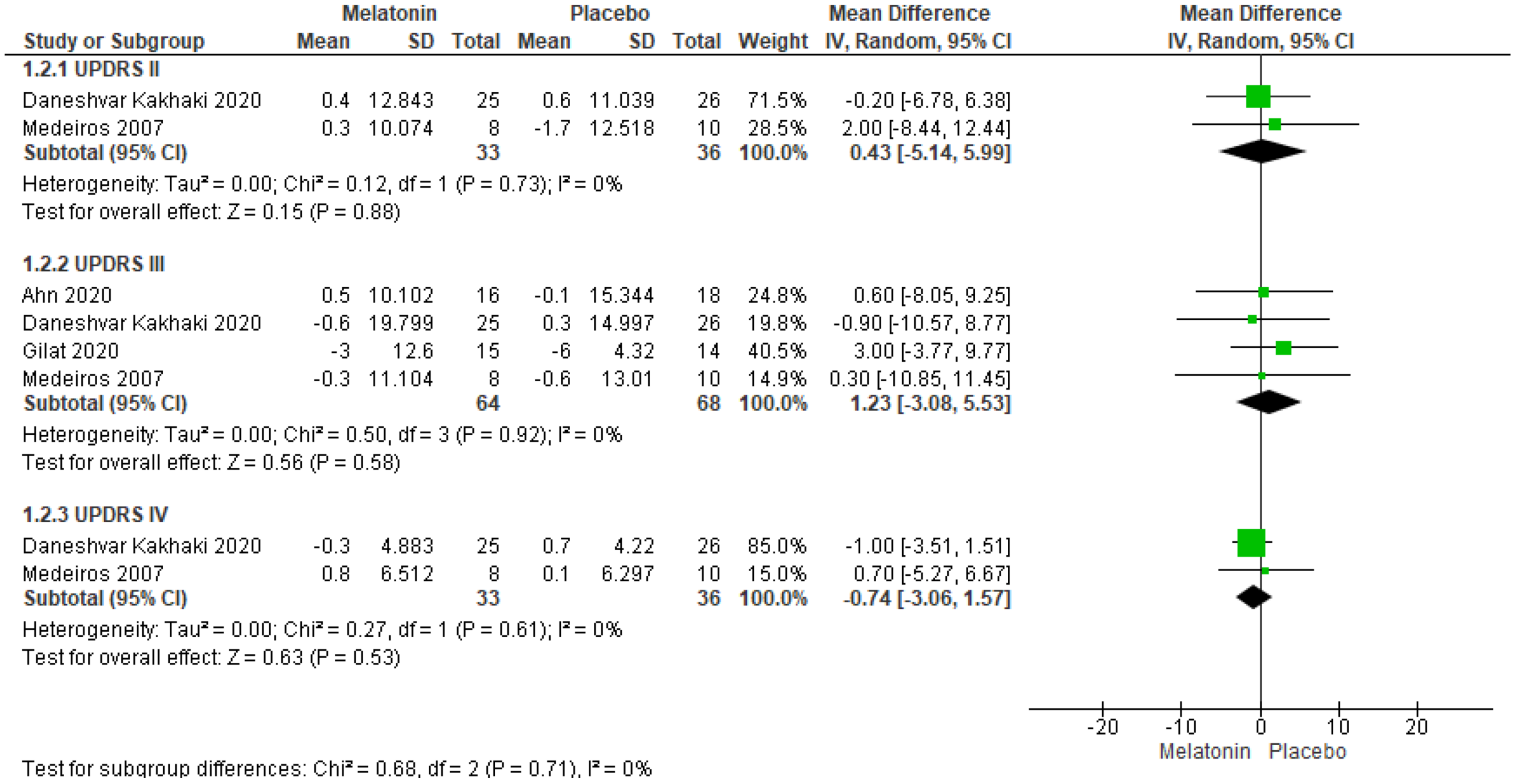
Forest plot comparing effects of exogenous melatonin on UPDRS subscale II, III and IV.

UPDRS subscale score were reported in 4 trials encompassing 132 patients (57–60) and analysis revealed that melatonin had no significant effect on individual UPDRS II, III, and IV scores, regardless of dosage and treatment duration, although a marginal reduction in UPDRS IV scores was observed favoring melatonin. Mean difference for each subscales were 0.43 (95% CI: −5.14 to 5.99, I^2^ = 0%, *p* = 0.88), 1.11 (95% CI: −3.08 to 5.53, I^2^ = 0%, *p* = 0.58) and − 0.74 (95% CI: −3.06 to 1.57, I^2^ = 0%, *p* = 0.53), respectively (Figure 4).

#### 3.4.2. Sleep quality

**Figure 5:**
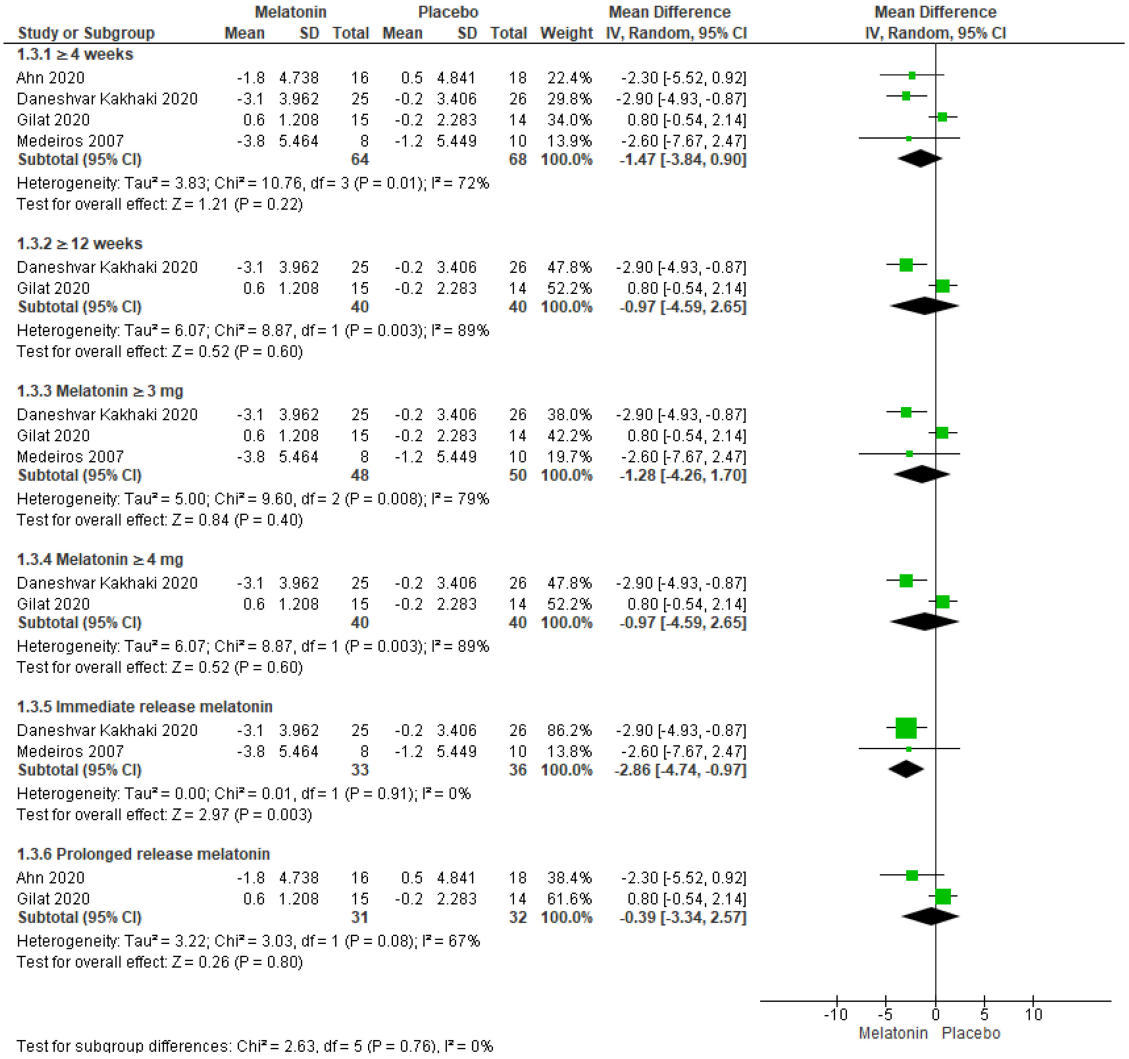
Forest plot comparing effects of exogenous melatonin on POSI score.

Subjective sleep quality was assessed using the PQSI scale in four trials including a total of 132 patients (57–60). No significant improvement in PQSI scores was observed in groups receiving melatonin for ≥4 weeks (57–60) (MD = −1.47, 95% CI: −3.84 to 0.90, I^2^ = 72%, *p* = 0.22) and ≥ 12 weeks (57, 58) (MD = −0.97, 95% CI: −4.59 to 2.65, I^2^ = 89%, *p* = 0.60, Figure 5). Heterogeneity in the ≥4 weeks cohort (I^2^ = 72%) was mitigated by the exclusion of the Gilat et al. trial, significantly altering the results in favor of melatonin (MD = -2.72, 95% CI: −4.34 to −1.09, I^2^ = 0%, *p* = 0.001) (Supplementary Table S3). However. inclusion of only two studies in ≥12 weeks cohort (I^2^ = 89%) limited its sensitivity analysis.

Further analysis based on dosage revealed no significant improvements with Melatonin ≥3 mg/day (57–59) (MD = −1.28, 95% CI: −4.26 to 1.70, I^2^ = 79%, *p* = 0.60) and Melatonin ≥4 mg/day (57, 58) (MD = −0.97, 95% CI: −4.59 to 2.65, I^2^ = 89%, *p* = 0.60, Figure 5). Sensitivity analysis in Melatonin ≥3 mg/day cohort (I^2^ = 79%) eliminated heterogeneity upon removing Gilat et al. trial, and the results significantly favored melatonin (MD = -2.86, 95% CI: −4.74 to −0.97, I^2^ = 0%, *p* = 0.003). On the other hand, sensitivity analysis on Melatonin ≥4 mg/day cohort (I^2^ = 89%), which included only two studies, was not performed.

In an exclusive analysis of trials utilizing immediate-release formulations (57, 59) melatonin showed statistically significant improvement in PQSI scores (MD = −2.86, 95% CI: −4.74 to −0.97, I^2^ = 0%, p = 0.003, Figure 5). In contrast, analysis of trials using prolonged-release formulations (58, 60) demonstrated no statistically significant improvement and high heterogeneity (MD = −0.39, 95% CI: −3.34 to −2.57, I^2^ = 67%, *p* = 0.80), however, the inclusion of only two studies limited the scope of sensitivity analysis.

### 3.5. Trial sequence analysis

**Figure 6:**
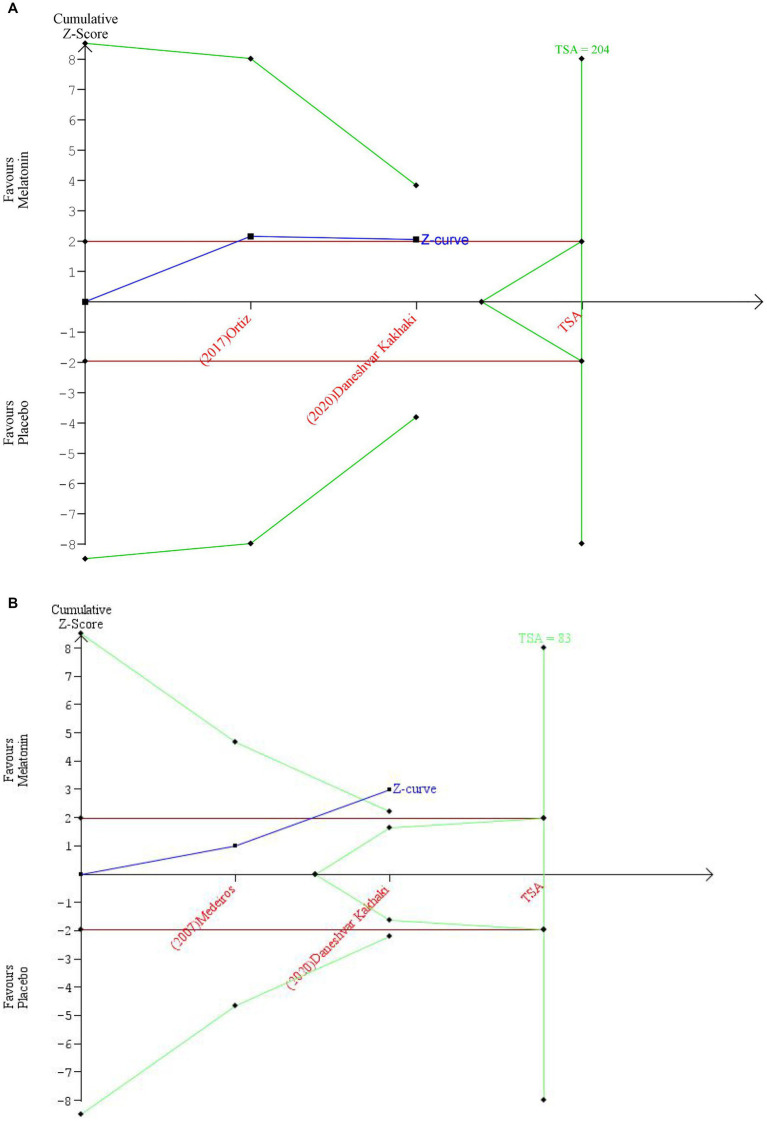
**(A)** Trial sequential analysis of the efficacy of melatonin vs. placebo on severity of Parkinson’s disease symptoms. **(B)** Trial sequential analysis of the efficacy of melatonin vs. placebo on sleep quality in Parkinson’s disease.

Trial Sequential Analysis on UPDRS total scores in Melatonin ≥10 mg/day group (Figure 6A) revealed that the cumulative z-score line did not cross any boundaries and remained within the “non-significant” zone, indicating insufficient statistical evidence. Additionally, Trial Sequential Analysis on PQSI scores in immediate release formulation group (Figure 6B) showed that cumulated z-score line did cross the benefit boundary, but the required information size was not reached. Therefore, despite observation of significant results, it is not yet appropriate to make direct clinical recommendations without further investigation.

### 3.6. Assessment of certainty of evidence

The GRADE assessment revealed low quality of evidence for UPDRS total scores and moderate quality of evidence for UPDRS II scores. However, evidence for UPDRS III, UPDRS IV, and PQSI scores was of high quality. Factors contributing to downgrading of evidence included high heterogeneity, wide confidence intervals, imprecision due to unavailable SDs, and insufficient directionality. The detailed GRADE summary of findings is provided in Supplementary Table S4.

## 4. Discussion

Our comprehensive meta-analysis indicates that melatonin therapy merits serious consideration for Parkinson’s disease. Dosages of ≥10 mg/day for a minimum duration of ≥12 weeks in immediate-release formulations, have consistently demonstrated significant potential. These findings reinforce the rationale of our study, suggesting that melatonin, when used in specific treatment regimens, may alleviate symptom severity and reduce sleep disturbances in Parkinson’s disease.

To the best of our knowledge, this is the first meta-analysis to date that evaluates efficacy of melatonin on motor symptoms in Parkinson disease using the standard Unified Parkinson Disease Rating Scale (UPDRS) (45). Analysis of UPDRS total scores indicate that after at least 12 weeks of treatment, melatonin significantly impacts Parkinson’s disease progression when doses of ≥10 mg/day are used. This trend of enhanced melatonin efficacy with higher doses at longer treatment durations has been consistently reported in trails comparing 50 mg/day melatonin with 0.25 mg (61, 62) and 50 mg/day melatonin with 5 mg (63) for various outcomes. Furthermore, trials included in our analysis also reported significant results with 50 mg/day melatonin for 1 year (56) and non-significant results with 10 mg/day or 4 mg/day melatonin for 12 weeks (57, 58). These findings, supported further by melatonin’s ability to exhibit virtually no acute or chronic toxicity (64, 65), strongly advocate its long-term utilization at higher doses as a safe choice.

Furthermore, it is important to address the observed heterogeneity in treatment response at different melatonin doses. Analysis of UPDRS total scores in groups receiving melatonin ≥10 mg/day revealed significant results with no heterogeneity (I^2^ = 0%). However, including studies with <10 mg doses increased heterogeneity substantially (I^2^ = 63%). Potential contributors may include dose-dependent and formulation-dependent pharmacokinetics of melatonin, as low dose studies used prolonged release formulations and high dose studies used immediate release formulations (44). Moreover, variations in treatment duration could also play a role, as longer durations with higher doses consistently demonstrated enhanced efficacy in previous studies (56–58).

Apart from dosage and duration, a crucial difference among these trails was the timing of melatonin intervention. In the significant study (56), melatonin was initiated in newly diagnosed patients immediately after observing a satisfactory response to anti-Parkinson’s therapy. In contrast, patients in non-significant studies (57, 58) had mean disease duration of 5.7 ± 1.9 and 5.0 ± 3.9 years respectively, indicating significant pre-existing damage at the time of melatonin introduction. This selection of patients with longer disease duration and introduction of melatonin at a later stage reveal an inherent flaw, as starting melatonin before neuronal loss is crucial for its free radical scavenging and antioxidant properties (18, 19, 31, 33, 42) to effectively prevent degeneration and reduce symptom severity in Parkinson’s disease. In addition, a sub-analysis focusing on only immediate-release formulations, also yielded significant results, however, use of prolonged-release formulation in only one study (58) hinders appropriate comparisons.

Taking a step further, we analyzed melatonin’s impact on each UPDRS subscale. Unfortunately, only one trial (57) reported UPDRS I score, rendering further analysis unfeasible, nevertheless, the results were statistically significant. Subsequent analysis indicated that melatonin has no significant effect on individual UPDRS II, III, and IV subscale scores, but it is also important to consider that these trials (57–60), included patients with average disease duration of >5 years, indicating significant pre-existing neuronal loss. Despite this, a slight reduction in UPDRS IV scores favored melatonin, possibly due to its ability to enhance efficacy and reduce side effects of other drugs through its anti-oxidant properties (15). This finding suggests that melatonin’s influence on UPDRS total scores may stem from its ability to counteract adverse treatment effects of Anti-Parkinson therapy (dyskinesias, off duration, nausea, vomiting, anorexia, insomnia, hypersomnia, orthostasis) (66), thereby enhancing overall treatment tolerability and quality of life in patients with Parkinson’s disease.

Lastly, we analyzed melatonin’s efficacy in improving sleep quality using the Pittsburgh Sleep Quality Index (PSQI) (47). Analysis revealed that melatonin will significantly improve PQSI scores only if immediate release formulations were used. Treatment durations of 4 and 12 weeks, doses of ≥3 and ≥ 4 mg/day, and prolonged-release formulations showed no significant impact, however, a significant heterogeneity was observed in these sub-analyzes. Excluding the Gilat et al. trial eliminated this heterogeneity, and the pooled estimates than favored melatonin for ≥4 weeks cohort (*p* = 0.001) and Melatonin ≥3 mg/day cohort (*p* = 0.003). This heterogeneity and its elimination can be attributed to the use of prolonged-release melatonin (PRM) in the excluded trial. PRM has been favored for sleep disorders by numerous case reports and small open label trials (67–73) as it mimics a more physiologic release (74), but has consistently failed to demonstrate a significant potential. It is also worth mentioning here that in our study, UPDRS scores analysis favored higher melatonin doses for longer durations, while PQSI scores analysis did not show a similar trend. This disparity can be explained by notorious sleep-inducing effects of melatonin, which are observed even at doses as low as 3 mg/day (75), and suggest melatonin’s pleiotropy (bifunctional pharmacology) in Parkinson’s disease.

In our analysis, we observed that melatonin supplementation in Parkinson’s disease demonstrated positive effects on both motor symptoms and sleep disturbances. However, sleep alone can directly influence motor symptoms too. The “sleep benefit,” an improvement of motor functions upon awakening, is seen in more than 40% of Parkinson’s disease patients, and is attributed to improved dopaminergic function as a result of increased storage of dopamine in nigrostriatal terminals during sleep (76). Hence, melatonin can indirectly lead to an improvement in motor symptoms through sleep improvement. This effect appears to be unrelated to its antioxidant properties, indicating a multifaceted potential for melatonin in Parkinson’s disease treatment.

We believe that our findings open up new avenues for clinicians and researchers to debate and explore in this topic. As far as we know, a systematic categorization of melatonin into dose groups for motor symptoms and sleep disturbances in Parkinson’s disease has not been conducted before, and is a defining feature of this meta-analysis. Furthermore, it strongly recommends the use of long-term, high-dose immediate-release melatonin in future investigations and emphasizes the significance of selecting patients with shorter disease duration and initiating melatonin early to fully explore its true therapeutic potential.

### 4.1. Limitations

Limitations of this meta-analysis stem from scarcity of available data, primarily due to the novelty of this topic. Only five RCTs, each with a small sample size, met the inclusion criteria and data from crossover RCTs were excluded due to lack of pre-crossover result reporting. Out of the 5 included trials, only 3 trials reported UPDRS total scores, thus highlighting the need of standardized reporting in future trials. Inconsistencies in reporting UPDRS scores and use of estimations for missing means and SDs in some studies further contribute to its limitations. Moreover, included studies only reported UPDRS score for motor outcomes; data on DAT imaging and other motor outcome assessment scales like H-Y stage were not available. Lastly, considerable heterogeneity was observed, particularly due to study by Gilat et al. While the exclusion of this outlier effectively eliminated heterogeneity, it is important to consider these limitations in the interpretation of findings.

### 4.2. Conclusion

Melatonin at dose of ≥10 mg/day for a minimum duration of ≥12 weeks and immediate-release formulations consistently demonstrated significant potential. Given its extensive range of effects, melatonin holds promise in improving both motor symptom and sleep disturbances in Parkinson disease. However, further trials are warranted to investigate its impact when initiated early in the disease course.

## Data availability statement

The original contributions presented in the study are included in the article/Supplementary material, further inquiries can be directed to the corresponding author.

## Author contributions

SI: Conceptualization, Methodology, Supervision, Validation, Visualization, Writing – review & editing. HS: Data curation, Formal analysis, Investigation, Resources, Software, Writing – original draft. Zainab: Data curation, Formal analysis, Investigation, Resources, Software, Writing – original draft.
