# Supplementary material for: Significant potential of melatonin therapy in Parkinson’s disease – a meta-analysis of randomized controlled trials
Source: Front Neurol. 2023 Oct 10;14:1265789. doi: 10.3389/fneur.2023.1265789 (PMC10597669; doi:10.3389/fneur.2023.1265789)
Supplement: Supplementary file 1 [file Table_1.DOCX]

Supplementary Material

Significant Potential of Melatonin Therapy in Parkinson's Disease – A Meta-analysis of Randomized Controlled Trials.

| **Table of Contents** | | |
| --- | --- | --- |
| Title | Content | Page |
| Table S1 | Literature search strategy | 2-3 |
| Table S2 | Sensitivity analysis of studies reporting UPDRS total scores | 4 |
| Table S3 | Sensitivity analysis of studies reporting PQSI scores | 4 |
| Table S4 | GRADEpro Quality of evidence Summary | 5 |
| Table S5 | PRISMA 2020 checklist | 6-8 |

**Table S1.** Literature search strategy (6/10/2023).

| **Concept 1: Melatonin Therapy** | | |
| --- | --- | --- |
| **Keywords:** “Melatonin” OR “melatonin therapy” OR “melatonin supplement*” OR “melatonin treatment*” OR “melatonin receptor agonist*” OR “effect of melatonin” OR “add-on melatonin”  **MeSH:** "Melatonin"[Mesh]  **Search Strategy:** "melatonin"[Mesh] OR “melatonin therapy” OR “melatonin supplement*” OR “melatonin treatment*” OR “melatonin receptor agonist*” OR “effect of melatonin” OR “add-on melatonin” | | |
| **Concept 2: Parkinson's disease** | | |
| **Keywords:** “Parkinson disease*” OR “Parkinson's disease*” OR “Parkinson's disorder*” OR “Parkinsonism” OR “Parkinson's symptom*” AND “Randomized Controlled Trials as Topic” OR “Meta-analysis”  **MeSH:** ("Parkinson disease"[Mesh]) AND ("Randomized Controlled Trials as Topic"[Mesh])  **Search Strategy:** ("Parkinson disease"[Mesh]) OR "Parkinson disease*” OR “Parkinson's disease*” OR “Parkinson's disorder*” OR “Parkinsonism” OR “Parkinson's symptom*”) AND (“Randomized Controlled Trials as Topic”[Mesh] OR "random allocation" OR "single-blind method" OR "double-blind method" OR "triple-blind method" OR "controlled clinical trial") AND (“meta-analysis” OR “systematic review”) | | |
| **Search Strategy in PubMed (65 articles)** | | |
| **Link:** <https://pubmed.ncbi.nlm.nih.gov/advanced/>  ("Melatonin"[MeSH Terms] AND "Parkinson Disease"[MeSH Terms]) AND (clinicaltrial[Filter] OR meta-analysis[Filter] OR randomizedcontrolledtrial[Filter] OR review[Filter] OR systematicreview[Filter]) | | |
| **Search Strategy in Cochrane (13 reviews, 26 trials, 2 protocols)** | | |
| **Link:** <https://www.cochranelibrary.com/advanced-search?cookiesEnabled>  Search Name: Parkinson Disease  Date Run: 06/10/2023 | | |
| **ID** | **Search Hits** | **Results** |
| #1 | MeSH descriptor: [Melatonin] explode all trees | 1505 |
| #2 | melatonin therapy (Word variations have been searched) | 1576 |
| #3 | melatonin supplement (Word variations have been searched) | 506 |
| #4 | melatonin treatment (Word variations have been searched) | 1946 |
| #5 | melatonin receptor agonist (Word variations have been searched) | 185 |
| #6 | MeSH descriptor: [Parkinson Disease] explode all trees | 6120 |
| #7 | Parkinson’s disease (Word variations have been searched) | 13080 |
| #8 | Parkinson’s disorder (Word variations have been searched) | 5936 |
| #9 | Parkinsonism (Word variations have been searched) | 13861 |
| #10 | Parkinson’s symptoms (Word variations have been searched) | 4488 |
| #11 | #1 OR #2 OR #3 OR #4 OR #5 | 2951 |
| #12 | #6 OR #7 OR #8 OR #9 OR #10 | 13863 |
| #13 | #10 AND #11 (in Cochrane Reviews and Trials) | 41 |
| **Search Strategy in ResearchGate (998 studies)** | | |
| **Link:** <https://www.researchgate.net/>  Keyword: Melatonin, clinical trials involving humans | | |
| **Search Strategy in Google Scholar (170 articles)** | | |
| **Link:** <https://scholar.google.com/> (Advance search box)  ("Melatonin Therapy") AND ("Parkinson disease" OR "Parkinson disorder" OR “Parkinsonism”)  Find articles:   - with all of the words: Melatonin therapy, Parkinson disease, human - with the exact phrase: Melatonin therapy - with at least one of the words: controlled trial, meta-analysis - without the words: Animal - where my words occur: anywhere in the article | | |
| **Search Strategy in Europe PMC (203 studies)** | | |
| **Link:** <https://europepmc.org/advancesearch>  Keyword: Melatonin,  Publication Type: Clinal trial, Review, Meta-analysis | | |
| **Search Strategy in WHO International Clinical Trials Registry Platform (ICTRP) (47 studies)** | | |
| **Link:** <https://trialsearch.who.int/AdvSearch.aspx>  Melatonin in the intervention  Recruitment status is: All  With results only | | |
| **Search Strategy in SciELO (76 studies)** | | |
| **Link:**<https://search.scielo.org/?q=*:*&lang=pt&count=15&from=0&output=site&sort=&format=summary&fb=&page=1&q=*&lang=pt&page=1>  Keyword: Melatonin  Filter: Medicine | | |
| **Search Strategy in ClinicalTrials.gov (6 studies)** | | |
| **Link:**<https://clinicaltrials.gov/ct2/search/advanced?cond=&term=&cntry=&state=&city=&dist=>  Condition or disease: Nervous System Diseases  Other terms: Melatonin  Study type: Interventional Studies (Clinical Trials)  Study results: Studies with results  Intervention/treatment: Melatonin | | |

**Supplementary Table S2:** Sensitivity analysis of studies reporting UPDRS total scores

|  | **Random-effects models** | |  | **Heterogeneity** | |
| --- | --- | --- | --- | --- | --- |
| **Study excluded** | **MD** | **95% Cl** |  | **I^2^ (%)** | **p Value** |
| Daneshvar Kakhaki 2020 | -2.02 | -28.15 to 24.11 |  | 82% | 0.02 |
| Ortiz 2017 | 3.89 | -12.56 to -1.18 |  | 37% | 0.21 |
| Gilat 2020 | -11.35 | -22.35 to -0.35 |  | 0% | 0.40 |
| MD = Mean Difference, CI = Confidence internal | | | | | |

**Supplementary Table S3:** Sensitivity analysis of studies reporting PQSI scores

|  | **Random-effects models** | |  | **Heterogeneity** | |
| --- | --- | --- | --- | --- | --- |
| **Study excluded** | **MD** | **95% Cl** |  | **I^2^ (%)** | **p Value** |
| Medeiros 2007 | -1.31 | -4.02 to1.41 |  | 80% | 0.006 |
| Ahn 2020 | -1.28 | -4.26 to 1.70 |  | 79% | 0.008 |
| Daneshvar Kakhaki 2020 | -2.60 | -7.67 to 2.47 |  | 53% | 0.12 |
| Gilat 2020 | --2.72 | -4.34 to -1.09 |  | 0% | 0.95 |
| MD = Mean Difference, CI = Confidence internal | | | | | |

**Table S2.** GRADEpro Quality of evidence Summary

| **Melatonin compared to Placebo for Parkinson disease** | | | | | |
| --- | --- | --- | --- | --- | --- |
| **Patient or population:** Parkinson disease  **Setting:** Inpatient/outpatient  **Intervention:** Melatonin  **Comparison:** Placebo | | | | | |
| **Outcomes** | **№ of participants (studies) Follow-up** | **Certainty of the evidence (GRADE)** | **Relative effect (95% CI)** | **Anticipated absolute effects** | |
|  |  |  |  | **Risk with Placebo** | **Risk difference with Melatonin** |
| Activities of Daily Living (UPDRS II) assessed with: UPDRS II scale Scale from: 0 to 52 follow-up: range 4 weeks to 12 weeks | 69 (2 RCTs) | ⨁⨁⨁◯ Moderate^a,b^ | - | The mean activities of Daily Living ranged from **13.8 to 21.3** points | MD **0.43 points higher** (5.14 lower to 5.99 higher) |
| Motor Examination (UPDRS III) assessed with: UPDRS III scale Scale from: 0 to 108 follow-up: range 4 weeks to 12 weeks | 132 (3 RCTs) | ⨁⨁⨁⨁ High^a,c^ | - | The mean motor Examination ranged from **15.7 to 31.1** points | MD **1.23 points higher** (3.08 lower to 5.53 higher) |
| Complications of Therapy (UPDRS IV) assessed with: UPDRS IV scale Scale from: 0 to 23 follow-up: range 4 weeks to 12 weeks | 69 (2 RCTs) | ⨁⨁⨁⨁ High^a,c^ | - | The mean complications of Therapy ranged from **3.4 to 6.0** points | MD **0.74 points lower** (3.06 lower to 1.57 higher) |
| Severity of Parkinson's disease symptoms (UPDRS total) assessed with: UPDRS I, II, III and IV scale Scale from: 0 to 200 follow-up: range 4 weeks to 52 weeks | 93 (3 RCTs) | ⨁⨁◯◯ Low^a,c,d,e^ | - | The mean severity of Parkinson's disease symptoms ranged from **64.5 to 71.1** Points | MD **3.15 Points higher** (23.72 lower to 30.02 higher) |
| Sleep quality (PQSI) assessed with: PQSI scale Scale from: 0 to 21 follow-up: range 4 weeks to 12 weeks | 103 (4 RCTs) | ⨁⨁⨁⨁ High^c,d^ | - | The mean sleep quality ranged from **4.5 to 7.7** Points | MD **1.47 Points lower** (3.84 lower to 0.9 higher) |
| ***The risk in the intervention group** (and its 95% confidence interval) is based on the assumed risk in the comparison group and the **relative effect** of the intervention (and its 95% CI). **CI:** confidence interval; **MD:** mean difference | | | | | |
| **GRADE Working Group grades of evidence** **High certainty:** we are very confident that the true effect lies close to that of the estimate of the effect. **Moderate certainty:** we are moderately confident in the effect estimate: the true effect is likely to be close to the estimate of the effect, but there is a possibility that it is substantially different. **Low certainty:** our confidence in the effect estimate is limited: the true effect may be substantially different from the estimate of the effect. **Very low certainty:** we have very little confidence in the effect estimate: the true effect is likely to be substantially different from the estimate of effect. | | | | | |

#### Explanations

Wide confidence interval indicates uncertainty and lack of precision in the estimate.

mean difference between -0.5 and 0.5

mean difference greater than 0.5 or less than -0.5

Heterogeneity (I2>50%) across studies

Downgraded by 1 as outcome was not sufficiently directed in one direction

| **Section and Topic** | **Item #** | **Checklist item** | **Location where item is reported** |
| --- | --- | --- | --- |
| **TITLE** | | |  |
| Title | 1 | Identify the report as a systematic review. | Page 1 |
| **ABSTRACT** | | |  |
| Abstract | 2 | See the PRISMA 2020 for Abstracts checklist. | Page 1 |
| **INTRODUCTION** | | |  |
| Rationale | 3 | Describe the rationale for the review in the context of existing knowledge. | Section 2 |
| Objectives | 4 | Provide an explicit statement of the objective(s) or question(s) the review addresses. | Section 2 |
| **METHODS** | | |  |
| Eligibility criteria | 5 | Specify the inclusion and exclusion criteria for the review and how studies were grouped for the syntheses. | Section 3.2 |
| Information sources | 6 | Specify all databases, registers, websites, organisations, reference lists and other sources searched or consulted to identify studies. Specify the date when each source was last searched or consulted. | Section 3.1 |
| Search strategy | 7 | Present the full search strategies for all databases, registers and websites, including any filters and limits used. | Table S1 |
| Selection process | 8 | Specify the methods used to decide whether a study met the inclusion criteria of the review, including how many reviewers screened each record and each report retrieved, whether they worked independently, and if applicable, details of automation tools used in the process. | Section 3.3 |
| Data collection process | 9 | Specify the methods used to collect data from reports, including how many reviewers collected data from each report, whether they worked independently, any processes for obtaining or confirming data from study investigators, and if applicable, details of automation tools used in the process. | Section 3.3 |
| Data items | 10a | List and define all outcomes for which data were sought. Specify whether all results that were compatible with each outcome domain in each study were sought (e.g. for all measures, time points, analyses), and if not, the methods used to decide which results to collect. | Section 3.3 |
|  | 10b | List and define all other variables for which data were sought (e.g. participant and intervention characteristics, funding sources). Describe any assumptions made about any missing or unclear information. | Section 3.3 |
| Study risk of bias assessment | 11 | Specify the methods used to assess risk of bias in the included studies, including details of the tool(s) used, how many reviewers assessed each study and whether they worked independently, and if applicable, details of automation tools used in the process. | Section 3.4 |
| Effect measures | 12 | Specify for each outcome the effect measure(s) (e.g. risk ratio, mean difference) used in the synthesis or presentation of results. | Section 3.6 |
| Synthesis methods | 13a | Describe the processes used to decide which studies were eligible for each synthesis (e.g. tabulating the study intervention characteristics and comparing against the planned groups for each synthesis (item #5)). | Section 3.3 |
|  | 13b | Describe any methods required to prepare the data for presentation or synthesis, such as handling of missing summary statistics, or data conversions. | Section 3.3 |
|  | 13c | Describe any methods used to tabulate or visually display results of individual studies and syntheses. | Section 3.3 |
|  | 13d | Describe any methods used to synthesize results and provide a rationale for the choice(s). If meta-analysis was performed, describe the model(s), method(s) to identify the presence and extent of statistical heterogeneity, and software package(s) used. | Section 3.6 |
|  | 13e | Describe any methods used to explore possible causes of heterogeneity among study results (e.g. subgroup analysis, meta-regression). | Section 3.6 |
|  | 13f | Describe any sensitivity analyses conducted to assess robustness of the synthesized results. | Section 3.6 |
| Reporting bias assessment | 14 | Describe any methods used to assess risk of bias due to missing results in a synthesis (arising from reporting biases). | Section 3.6 |
| Certainty assessment | 15 | Describe any methods used to assess certainty (or confidence) in the body of evidence for an outcome. | Section 3.5 |
| **RESULTS** | | |  |
| Study selection | 16a | Describe the results of the search and selection process, from the number of records identified in the search to the number of studies included in the review, ideally using a flow diagram. | Section 4.1 |
|  | 16b | Cite studies that might appear to meet the inclusion criteria, but which were excluded, and explain why they were excluded. | Section 4.1 |
| Study characteristics | 17 | Cite each included study and present its characteristics. | Section 4.2 and Table 1 |
| Risk of bias in studies | 18 | Present assessments of risk of bias for each included study. | Figure 2 |
| Results of individual studies | 19 | For all outcomes, present, for each study: (a) summary statistics for each group (where appropriate) and (b) an effect estimate and its precision (e.g. confidence/credible interval), ideally using structured tables or plots. | Section 4.4 |
| Results of syntheses | 20a | For each synthesis, briefly summarise the characteristics and risk of bias among contributing studies. | Section 4.3 |
|  | 20b | Present results of all statistical syntheses conducted. If meta-analysis was done, present for each the summary estimate and its precision (e.g. confidence/credible interval) and measures of statistical heterogeneity. If comparing groups, describe the direction of the effect. | Section 4.3 |
|  | 20c | Present results of all investigations of possible causes of heterogeneity among study results. | Section 4.3 |
|  | 20d | Present results of all sensitivity analyses conducted to assess the robustness of the synthesized results. | Section 4.3 |
| Reporting biases | 21 | Present assessments of risk of bias due to missing results (arising from reporting biases) for each synthesis assessed. | Section 4.5 |
| Certainty of evidence | 22 | Present assessments of certainty (or confidence) in the body of evidence for each outcome assessed. | Section 4.6 |
| **DISCUSSION** | | |  |
| Discussion | 23a | Provide a general interpretation of the results in the context of other evidence. | Section 5 |
|  | 23b | Discuss any limitations of the evidence included in the review. | Section 5.1 |
|  | 23c | Discuss any limitations of the review processes used. | Section 5.1 |
|  | 23d | Discuss implications of the results for practice, policy, and future research. | Section 5 |
| **OTHER INFORMATION** | | |  |
| Registration and protocol | 24a | Provide registration information for the review, including register name and registration number, or state that the review was not registered. | Section 3 |
|  | 24b | Indicate where the review protocol can be accessed, or state that a protocol was not prepared. | Section 3 |
|  | 24c | Describe and explain any amendments to information provided at registration or in the protocol. | NA |
| Support | 25 | Describe sources of financial or non-financial support for the review, and the role of the funders or sponsors in the review. | Page 7 |
| Competing interests | 26 | Declare any competing interests of review authors. | Page 7 |
| Availability of data, code and other materials | 27 | Report which of the following are publicly available and where they can be found: template data collection forms; data extracted from included studies; data used for all analyses; analytic code; any other materials used in the review. | Page 7 |

*From:*  Page MJ, McKenzie JE, Bossuyt PM, Boutron I, Hoffmann TC, Mulrow CD, et al. The PRISMA 2020 statement: an updated guideline for reporting systematic reviews. BMJ 2021;372:n71. doi: 10.1136/bmj.n71

For more information, visit: <http://www.prisma-statement.org/>
